# Association Between Type 1 Diabetes Mellitus and Eating Disorders: A Systematic Review and Meta‐Analysis

**DOI:** 10.1002/edm2.473

**Published:** 2024-04-10

**Authors:** Yomna E. Dean, Karam R. Motawea, Muaaz Aslam, Jose J. Loayza Pintado, Helen A. O. Popoola‐Samuel, Mohamed Salam, Prashant Obed Reddy Dundi, Webster Donaldy, Esraa M. Aledani, Zaineh Alqiqie, Nazia Sultana, Alaa Ramadan Hussein Mohamed, Amir Elalem, Sidra Tahreem Hashmi Syeda, Mai Saad Mohamed, Mazen W. Assal, Nada M. Attia, Hanan Hagar, Heba Ahmed Abdelaziz, Anuj Subedi, Areeg Elbahaie, Yusef Hazimeh, Hani Aiash

**Affiliations:** ^1^ Faculty of Medicine Alexandria University Alexandria Egypt; ^2^ Shaikh Khalifa Bin Zayed Al‐Nahyan Medical and Dental College Lahore Pakistan; ^3^ Universidad de San Martin de Porres Facultad de Medicina Humana Lima Peru; ^4^ College of Health and Sciences Rush University Chicago Illinois USA; ^5^ Mediclinic City Hospital Dubai UAE; ^6^ Karnataka Institute of Medical Sciences RGUHS Hubli India; ^7^ Harlem Hospital Center New York City New York USA; ^8^ Medical College University of Basra Basra Iraq; ^9^ Odessa National Medical University Odessa Ukraine; ^10^ Shadan Institute of Medical Sciences Hyderabad India; ^11^ Faculty of Medicine Suez Canal University Ismailia Egypt; ^12^ Deccan College of Medical Sciences Hyderabad India; ^13^ Faculty of Medicine Zagazig University Zagazig Egypt; ^14^ High Institute of Public Health Alexandria University Alexandria Egypt; ^15^ Prithvi Narayan Community Hospital Gorkha Nepal; ^16^ Ibn Sina National College Jeddah Saudi Arabia; ^17^ Lebanese University Beirut Lebanon; ^18^ SUNY Upstate Medical University Syracuse New York USA

**Keywords:** anorexia nervosa, bulimia nervosa, eating disorders, T1DM

## Abstract

**Background:**

Previous meta‐analyses have shown mixed results regarding the association between eating disorders (EDs) and type 1 diabetes mellitus (T1DM). Our paper aimed to analyse different EDs and disordered eating behaviours that may be practiced by patients with T1DM.

**Methods:**

A literature search of PubMed, Scopus and Web of Science was conducted on 17 January 2023, using the key terms “T1DM,” “Eating Disorders” and “Bulimia.” Only observational controlled studies were included. The Revman software (version 5.4) was used for the analysis.

**Results:**

T1DM was associated with increased risk of ED compared with nondiabetic individuals (RR = 2.47, 95% CI = 1.84–3.32, *p*‐value < 0.00001), especially bulimia nervosa (RR = 2.80, 95% CI = 1.18–6.65, *p*‐value = 0.02) and binge eating (RR = 1.53, 95% CI = 1.18–1.98, *p*‐value = 0.001). Our analysis has shown that increased risk of ED among T1DM persisted regardless of the questionnaire used to diagnose ED; DM‐validated questionnaires (RR = 2.80, 95% CI = 1.91–4.12, *p*‐value < 0.00001) and generic questionnaires (RR = 2.03, 95% CI = 1.27–3.23, *p*‐value = 0.003). Prevalence of insulin omission/misuse was 10.3%; diabetic females demonstrated a significantly higher risk of insulin omission and insulin misuse than diabetic males.

**Conclusion:**

Our study establishes a significant and clear connection between EDs and T1DM, particularly bulimia and binge eating, with T1DM. Moreover, female diabetics are at higher risk of insulin misuse/omission. Early proactive screening is essential and tailored; comprehensive interventions combining diabetes and ED components are recommended for this population, with referral to a specialised psychiatrist.

## Graphical abstract

Patients with T1DM had a higher risk of eating disorders than the control group. Specifically, bulimia nervosa and binge eating exhibited statistically significant associations with T1DM, while anorexia nervosa did not. The risk of disordered eating behaviours did not differ significantly between patients with T1DM and the control group. However, diabetic patients did exhibit a significantly elevated risk of insulin omission/misuse.

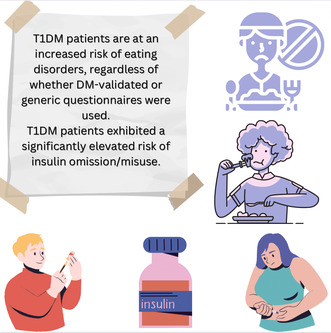

## 1 Introduction

Eating disorders (EDs) are characterised by excessive concern regarding body weight that results in unhealthy eating habits and behaviours [1]. A previous meta‐analysis conducted in 2005 has found that bulimia nervosa (BN), a type of ED, is significantly associated with type 1 diabetes mellitus (T1DM), while such an association was not seen between anorexia nervosa (AN) and T1DM [2]. On the contrary, a more recent meta‐analysis, conducted in 2013, has shown an insignificant association between ED and T1DM compared with the healthy controls [3]. The observed relationship between EDs and T1DM appears to result from a complex interplay between individual and environmental factors in the development of EDs. T1DM was linked to multiple risk factors for EDs such as higher BMI, low self‐esteem and dietary restraint. It is important to note that T1DM may practice several forms of disordered eating behaviours (DEBs), which are milder forms of EDs, such as unhealthy dieting and purging. Furthermore, patients with T1DM are treated via lifelong insulin administration which could adversely result in weight gain [4]. Recent studies have coined a new term, ‘Diabulimia’ which refers to the limitation or skipping of insulin doses by patients with T1DM, commonly observed among adolescents, with the objective of weight control [5]. This is further reinforced by De Paoli et al. findings, which concluded that insulin restriction is a DEB among patients with T1DM. Insulin omission could lead to devastating outcomes such as life‐threatening diabetic ketoacidosis and earlier onset of diabetic microvascular complications [6]. Other potential unhealthy behaviours practiced by patients with T1DM to control their weight include excessive exercise and diuretic abuse [7].

The presence of EDs in individuals with T1DM poses a significant health risk. It is associated with compromised metabolic control and approximately a threefold increase in the risk of diabetic retinopathy [8], along with adverse short‐term and long‐term physical consequences [9, 10]. Additionally, it has detrimental psychological effects, including lower psychosocial quality of life, diminished subjective well‐being and fewer effective coping strategies [11].

In 2005 and 2013, two meta‐analyses were conducted on this topic [2, 3]. Since then, multiple studies have been published; accordingly, we aimed to analyse the association between T1DM and EDs as well as different subtypes of EDs. Moreover, we aimed to study the potential DEBs practiced by patients with T1DM that could lead to harmful outcomes in the long run.

## 2 Methods

This study was conducted according to Preferred Reporting Items for Systematic reviews and Meta‐Analyses (PRISMA) guidelines [12], and its protocol was registered on Prospero (CRD42023392418).

### 2.1 Search Strategy

A literature search of the following databases: PubMed, Scopus and Web of Science on 17 January 2023, using key terms such as “T1DM,” “Eating Disorders” and “Bulimia,” was performed to identify the studies of interest (view Appendix S1 for the full search strategy).

### 2.2 Inclusion and Exclusion Criteria

We screened studies by titles and abstracts according to the following criteria:

Inclusion criteria: Cross‐sectional and controlled observational studies with data on the prevalence of EDs among patients with T1DM, including case–control and cohort studies. No restriction was made regarding the date of publication of the studies.

Exclusion criteria: Uncontrolled observational studies, editorials, letters to the editor, commentaries, reviews, systematic reviews, meta‐analyses, case reports, case series, animal studies and studies in a language other than English.

In the case of duplicate studies, the most recent study with the largest study population was included.

### 2.3 Study Selection

For each study, two independent co‐authors (P.R. and M.A.) reviewed the studies according to our criteria. If a consensus is not achieved, a third independent reviewer (Y.E.D.) was assigned to resolve the conflict.

### 2.4 Data Extraction and Quality Assessment

For each included study, two independent co‐authors (A.E. and W.D.) extracted the data according to our criteria. If a consensus is not achieved, a third independent reviewer (Y.E.D.) was assigned to resolve the conflict.

For the baseline and summary, the following data were extracted from the eligible studies relevant to the authors: first author's name, year of publication, country, study design, sample size; and relevant for the studies extracted: age, gender, BMI, duration of diabetes, HbA1c, age at onset of diabetes, type of ED questionnaire and a brief conclusion.

For the outcomes, the following data were extracted: EDs (assessment based on diabetes mellitus‐validated or general questionnaires such as Eating Attitude Test 26 or 40 [EAT], Assessment of Anorexia–Bulimia—Teenager version [BAB‐T], The Bulimic Investigatory Test, Edinburgh [BITE], Children's Depression Inventory [CDI], Children's Eating Disorder Examination [cEDE], The Assessing Health and Eating among Adolescents with Diabetes [AHEAD], The Eating Disorder Examination Questionnaire [EDE‐Q], Eating Disorder Inventory [EDI], The Problematic Eating Behavior Examination Questionnaire [PEBE‐Q] and Rating for Anorexia and Bulimia [RAB‐T]), AN, bulimia, binge eating, Dieting Subscale EAT 26, Bulimia Subscale EAT 26, BITE symptom subscale, BITE severity subscale, behaviours including regular/excessive exercise, vomiting, laxatives, diuretic misuse, binge eating, diet pills use and insulin omission/misuse.

Diabetes Mellitus‐Validated Eating Disorder Questionnaires are ED questionnaires that have been shown to be reliable and valid screening measures for EDs in both diabetic and nondiabetic populations. We have classified the questionnaires in a similar manner to the earlier meta‐analysis conducted in 2013 [3].

The risk of bias was assessed utilising Newcastle‐Ottawa Scale (NOS) items [13], with a total score of nine points, to evaluate the quality of observational studies. We defined the observational studies with a NOS score of ≥ 7 stars as high quality and NOS score of < 7 stars as low quality.

### 2.5 Data Analysis

Data were analysed by the RevMan software, version 5.4. Sensitivity analysis (leave‐one‐out test and subgroup analysis) was used. If no heterogeneity was observed, results were presented in a fixed effect model, and a random effect model if significant heterogeneity was observed. A relative risk (RR) with a 95% confidence interval (CI) was used to present dichotomous data, while mean difference (MD) with a 95% CI was used to present continuous data. Results were considered significant if the *p*‐value was < 0.05. Heterogeneity was defined as the variation or diversity in study outcomes among the studies included in the meta‐analysis. It may be due to different factors, such as the characteristics of the participants, study designs, the methods of analysis or the sources of bias [14].

## 3 Results

### 3.1 Literature Search

**FIGURE 1:**
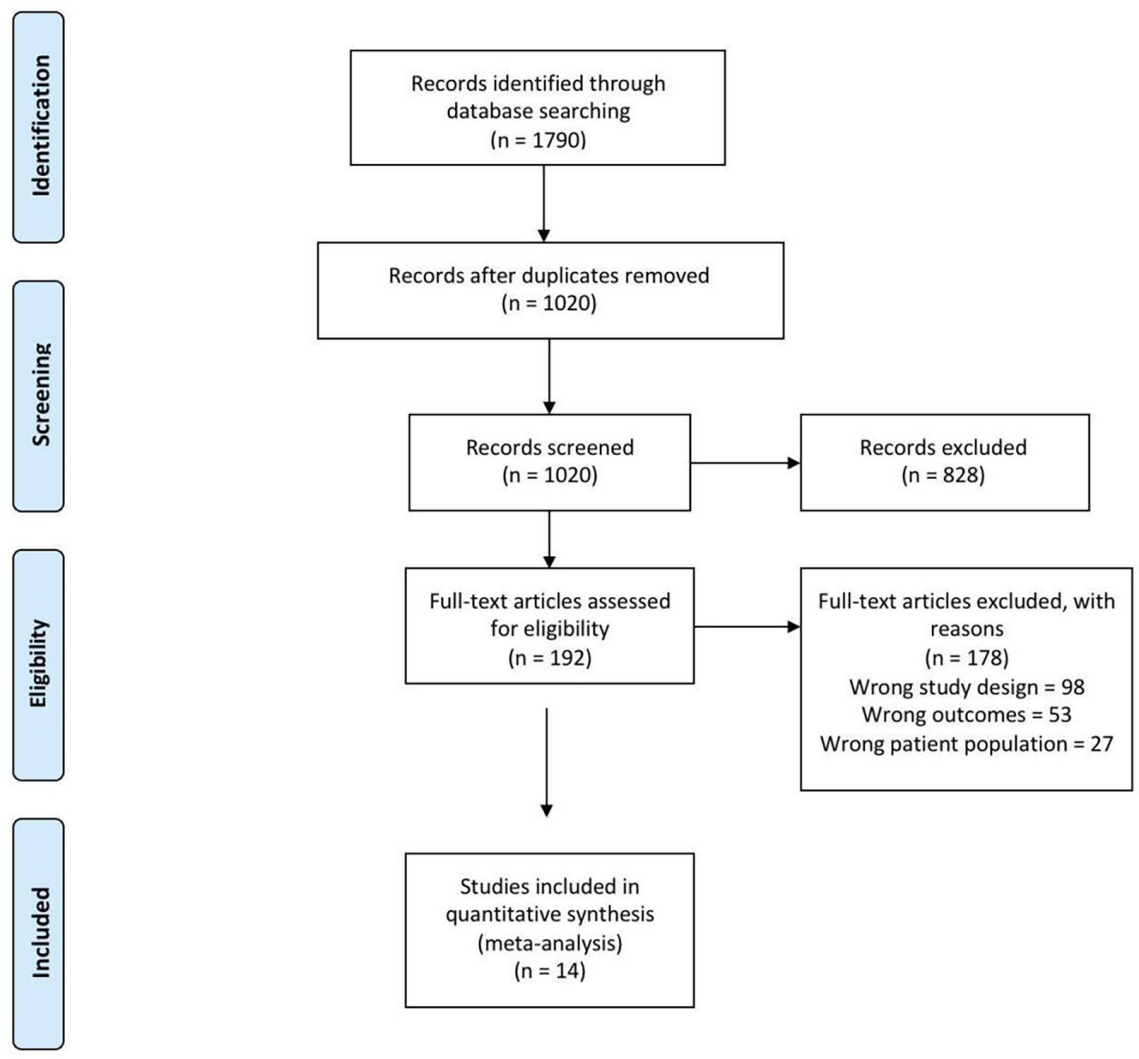
PRISMA flow diagram.

After a search of the PubMed, Scopus and Web of Science on 17 January 2023, 1790 studies resulted, of which 1020 studies were found eligible for title and abstract screening after the removal of duplicates. Of the 1020, 828 were irrelevant and 192 studies were eligible for full‐text screening. Finally, 14 studies [15, 16, 17, 18, 19, 20, 21, 22, 23, 24, 25, 26, 27, 28] were included in the meta‐analysis after full‐text screening, as shown in the PRISMA in Figure 1.

The total number of patients included in the study is 9079 patients, 1391 patients in the T1DM group and 7688 individuals in the control group; other baseline data are shown in Table 1. The quality assessment of the included studies is shown in Table 2.

**TABLE 1:** Baseline and summary of the included studies.

Author, year	Country	Study design	Sample size	Age, mean (SD)	Male, no. (%)	BMI, mean (SD)	Duration of diabetes	HBA1c	Age at onset of diabetes	Eating Disorder Questionnaire (e.g., EDE‐Q, etc.)	Generic or DM‐Validated Questionnaire	Conclusion
Ackard, 2008	USA	Case‐controlled	4889	14.91 (1.72)	2450 (50.11)	22.29 (4.49)	At least 12 months	8.8 (1.6)	No	AHEAD & EAT	Generic	Despite medical supervision, some TIDM adolescents showed disordered weight control behaviours including insulin misuse, which may cause complications
Broadley, 2019	Australia	Cross‐sectional	97	26.15 (7.0)	0	23.89 (4.34)	14.57 (7.74)	7.80 (1.33)	13.77 (8.18)	EDE‐Q	Generic	This study ties the high risk of developing DEB in T1DM in young females to their early focus on food‐related cues compared with the control group, which is important for future research
Troncone, 2019	Italy	Case‐controlled	108	12.73 (1.37)	48 (44.4)		7.23 (2.19)	8.01 (1.08)	4.72 (2.54)	PEBEQ	Generic	There is an association between T1DM and DEB; DEB is 33% higher in T1DM than in the control group, and its prevalence increases with age, weight and HbA1c. No gender bias. Parent evaluation helps in the early detection of any DEB
Smith, 2008	United Kingdom	Cross‐sectional	116	15.61 (1.43)	0	22.47 (3.43)				EDE‐Q	Generic	DEB is more frequent in the diabetics than in the control population
Jones, 2000	Canada	Cross‐sectional	1475	14.82 (1.92)	0	21.1 (3.54)	6.7 (3.6)	8.8 (1.7)	8.1 (3.6)	EAT‐26	DM‐validated	DSM‐IV and subthreshold eating disorders are almost twice as common in adolescent females with type 1 diabetes as in their nondiabetic peers
Roohafza, 2016	Iran	Cross‐sectional	435	16 (2.96)	0	20.87 (2.6)	At least 12 months			CDI & EAT‐26	DM‐validated	DEB is higher in diabetic girls. They have recorded higher scores in all three EAT‐26 subscales, mostly in dieting and bulimia
Robertson, 1990	Norway	Case‐controlled	116	27.9 (7.1)	0	22.69 (2.70)	13.1 (12.94)		13.1 (7.51)	EAT & BITE	Generic	This study does not support the hypothesis that DEB, mainly anorexia nervosa and bulimia nervosa, occurs more frequently in patients with IDDM. The scores on EAT and BITE were nonsignificant in both patients and controls
Fairburn, 1991	United Kingdom	Case‐controlled	167	21.27 (2.52)	46 (27.5)	23.64 (3.51)	9.6 (5.19)			EDE‐Q	Generic	This study showed no association between DEB and T1DM females
Yu‐Yun, 2009	Taiwan	Cross‐sectional	142	15.9 (3.1)	58 (41.9)	20.65 (3.2)	6.0 (3.7)	9.08 (1.96)	9.9 (3.6)	BITE & EAT‐26	DM‐validated	Both genders of T1DM adolescents showed higher prevalence of DEB
Friedman, 1997	France	Cross‐sectional	168	26.0 (6.9)	54 (32)	22.6 (2.5)	11.2 (6.9)	8.6 (1.7)	12.2 (5.9)	EAT & BITE	DM‐validated	The study highlighted the prevalence of DEB among diabetic women. Eating disorders are associated with impaired metabolic control
García‐Reyna, 2004	Spain	Cross‐sectional	669	13.73 (0.7)	378 (56.5)	21 (3.6)	1.5 (3.35)	8.6 (1.6)		EAT‐40 & EDEQ	Generic	EDNOS was found to be more common among male and female diabetic patients than in their nondiabetic counterparts
Engström, 1999	Sweden	Cross‐sectional	178	16.35 (1.4)	0	22.4 (3.2)	7.7 (3.8)	8.4 (2)		EDI & BAB‐T	DM‐validated	Compared with similar control subjects, girls diagnosed with IDDM exhibited a higher prevalence of eating disorders, primarily characterised by patterns of binge eating and self‐induced vomiting
Colton, 2004	Canada	Cross‐sectional	404	11.8 (1.5)	0	19.6 (3.6)	At least 6 months	8.2 (1)	7.1 (3)	cEDE	DM‐validated	Among preteen and early teenage girls, both with and without type 1 diabetes, there was a moderate prevalence of disturbed eating behaviour, typically of mild severity
Svensson, 2003	Sweden	Cross‐sectional	248	16.49 (1.1)	248 (100)	21.95 (3.1)	7.2 (4)	7.6 (1.5)		EDI‐C & RAB‐T	DM‐validated	The patients displayed notably elevated scores on the Drive for Thinness subscale in the EDI‐C when compared to the control subjects

Abbreviations: AHEAD = the Assessing Health and Eating among Adolescents with Diabetes, BAB‐T = Assessment of Anorexia–Bulimia—Teenager version, BITE = The Bulimic Investigatory Test, Edinburgh, BMI = body mass index, CDI = Children’s Depression Inventory, cEDE = Children’s Eating Disorder Examination, DEB = disordered eating behaviour, EAT = The Eating Attitude Test, ED = eating disorder, EDE‐Q = The Eating Disorder Examination Questionnaire, EDI = Eating Disorder Inventory, EDI‐C = Eating Disorder Inventory for Children, EDNOS = eating disorders not otherwise specified, IDDM = insulin‐dependent diabetes mellitus, PEBEQ = The Problematic Eating Behavior Examination Questionnaire, RAB‐T = Rating for Anorexia and Bulimia, SD = standard deviation, T1DM = type one diabetes mellitus.

**TABLE 2:** Quality assessment of the included studies.

Author, year	Selection				Comparability	Exposure		Total point
	(1) Representativeness of the exposed cohort	(2) Selection of the nonexposed	(3) Ascertainment of exposure	(4) Nonrespondents	(1) Comparability of cohorts on the basis of the design or analysis	(1) Assessment of outcome	(2) Statistical test	
Ackard, 2008	0	1	2	1	2	0	1	7
Broadly, 2019	1	1	1	0	2	0	1	6
Troncone, 2019	1	1	2	1	0	0	1	6
Smith, 2008	1	1	2	0	1	0	1	6
Jones, 2000	1	1	2	1	1	0	1	7
Roohafza, 2006	1	1	2	1	1	0	1	7
Robertson, 1990	1	1	2	0	1	0	0	5
Fairburn, 1991	0	1	2	0	0	0	1	4
Yu‐Yun, 2009	1	1	2	1	1	0	0	6
Friedman, 1997	1	1	2	0	0	0	1	5
García‐Reyna, 2004	1	1	2	1	0	0	0	5
Engström, 1999	1	1	2	1	1	0	1	7
Colton, 2004	1	1	2	0	1	1	1	7
Svensson, 2003	1	1	2	1	1	0	1	7

### 3.2 Outcomes

#### 3.2.1 Eating Disorder

**FIGURE 2:**
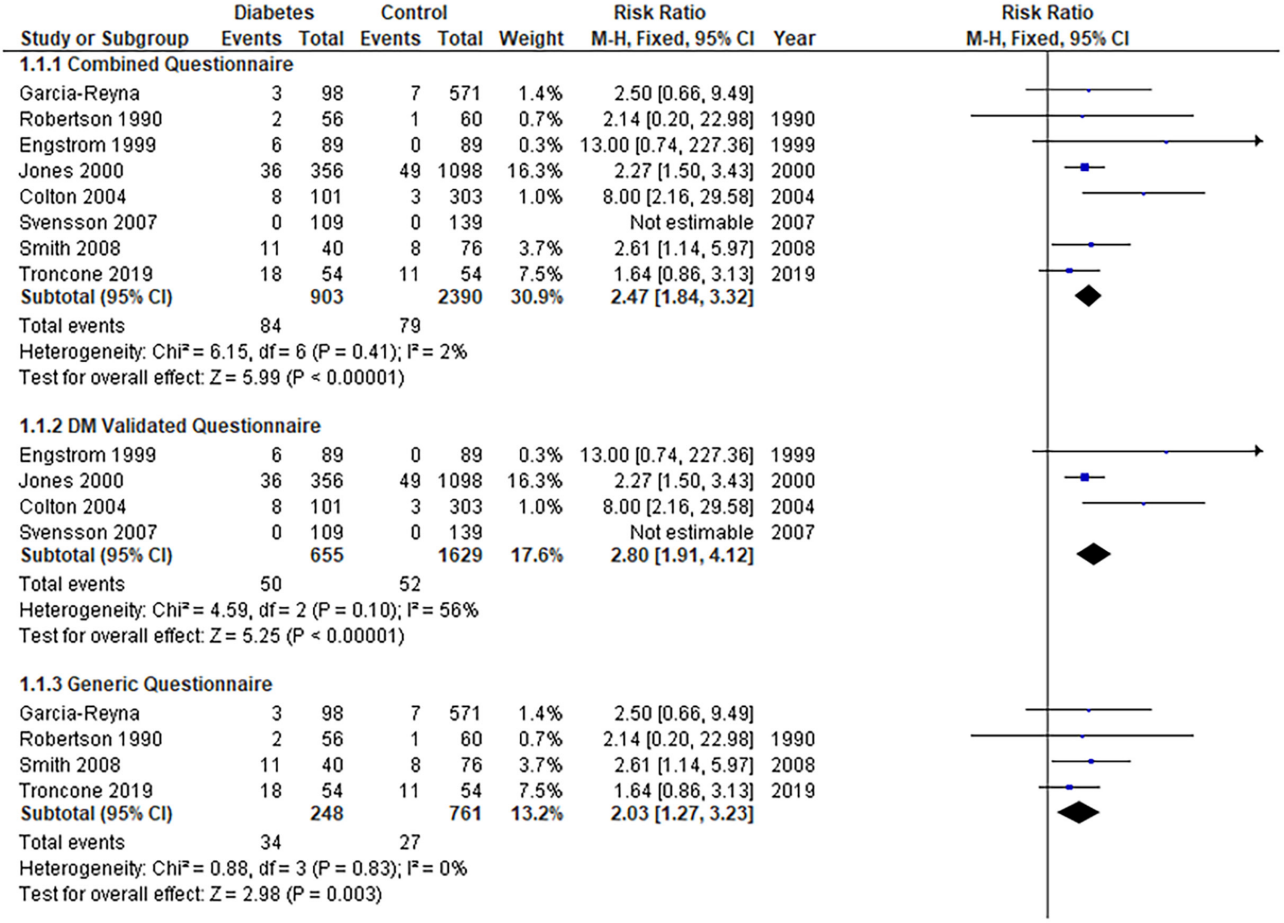
Eating disorder.

The pooled analysis showed a statistically significant association between the diabetes group and an increased incidence of ED compared with the control group (RR = 2.47, 95% CI = 1.84–3.32, *p*‐value < 0.00001). We observed no significant heterogeneity among studies (*p* = 0.41, *I* ^2^ = 2%) (Figure 2).

#### 3.2.2 Subgroups

##### 3.2.2.1 Eating Disorders DM‐Validated Questionnaire

The pooled analysis showed a statistically significant difference between the diabetes group and the control group (RR = 2.80, 95% CI = 1.91–4.12, *p*‐value < 0.00001). We observed no significant heterogeneity among studies (*p* = 0.10, *I* ^2^ = 56%) (Figure 2).

##### 3.2.2.2 Eating Disorders Generic Questionnaire

The pooled analysis showed a statistically significant association between the diabetes group and the control group (RR = 2.03, 95% CI = 1.27–3.23, *p*‐value = 0.003). We observed no significant heterogeneity among studies (*p* = 0.83, *I* ^2^ = 0%) (Figure 2).

#### 3.2.3 Anorexia Nervosa

**FIGURE 3:**
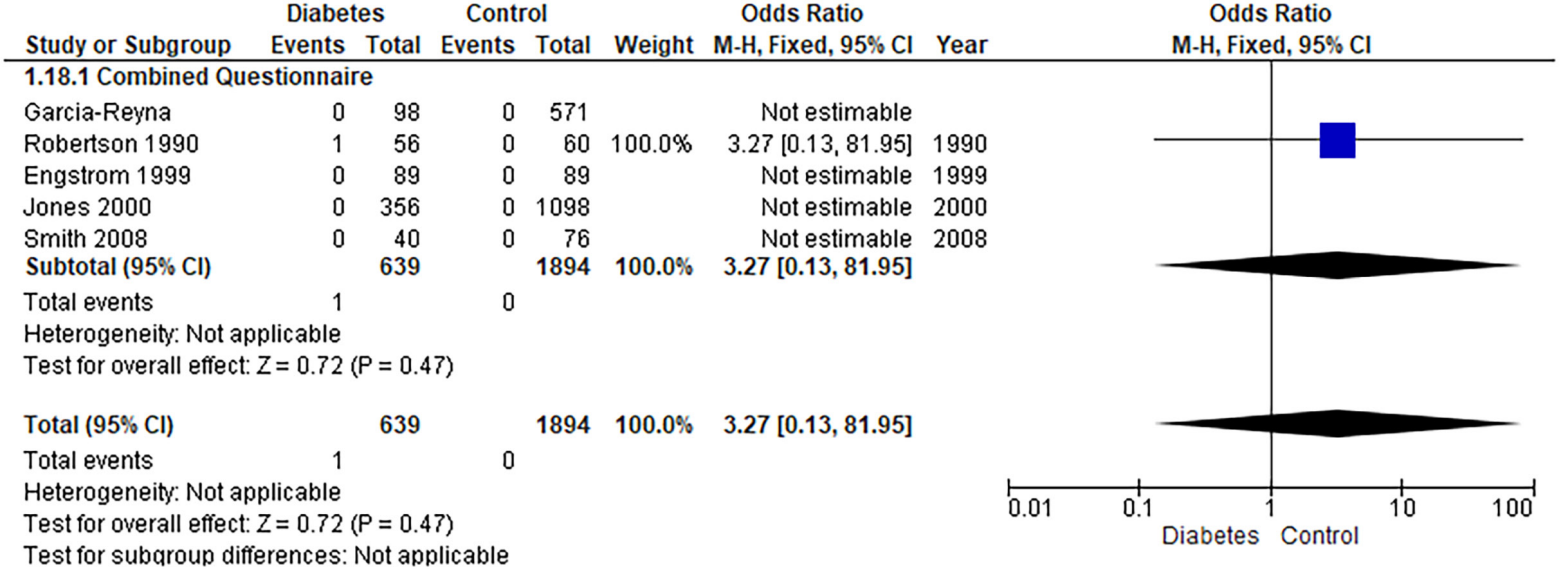
Anorexia nervosa.

The pooled analysis showed no statistically significant difference between the diabetes group and the control group (RR = 3.27, 95% CI = 0.13–81.95, *p*‐value = 0.47) (Figure 3).

#### 3.2.4 Bulimia Nervosa

**FIGURE 4:**
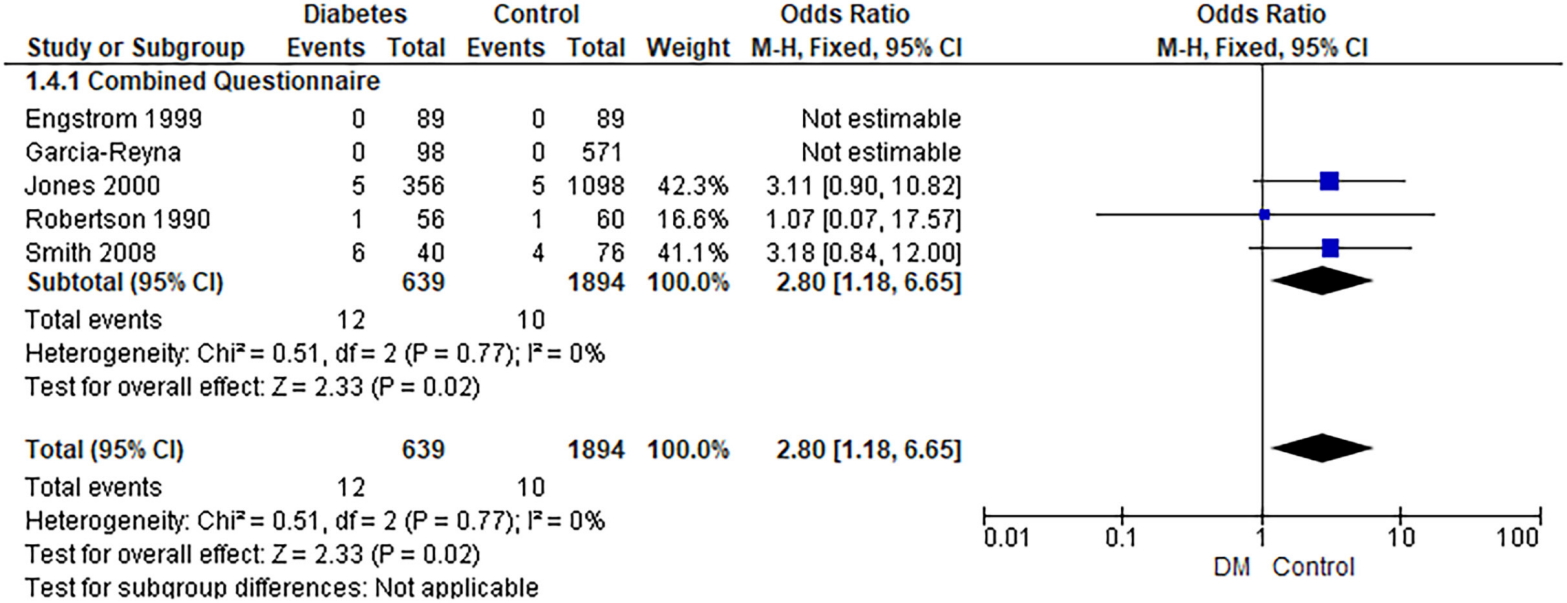
Bulimia nervosa.

**FIGURE 5:**
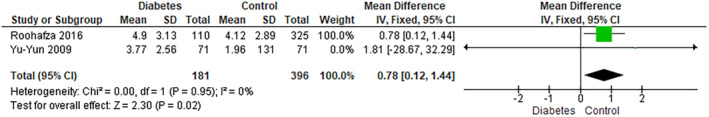
Bulimia subscale EAT 26.

The pooled analysis showed a statistically significant association between the diabetes group and an increased incidence of BN compared with the control group (RR = 2.80, 95% CI = 1.18–6.65, *p*‐value = 0.02). We observed no significant heterogeneity among studies (*p* = 0.77, *I* ^2^ = 0%) (Figure 4). In addition, the pooled analysis showed a statistically significant association between the diabetes group and increased Bulimia Subscale EAT 26 (MD = 0.78, 95% CI = 0.12–1.44, *p*‐value = 0.02). We observed no significant heterogeneity among studies (*p* = 0.95, *I* ^2^ = 0%) (Figure 5).

#### 3.2.5 Binge Eating

**FIGURE 6:**
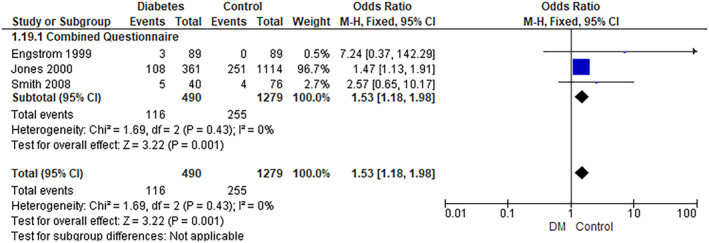
Binge eating.

The pooled analysis showed a statistically significant association between the diabetes group and an increased incidence of binge eating compared with the control group (RR = 1.53, 95% CI = 1.18–1.98, *p*‐value = 0.001). We observed no significant heterogeneity among studies (*p* = 0.43, *I* ^2^ = 0%) (Figure 6).

#### 3.2.6 Dieting Subscale EAT 26

**FIGURE 7:**
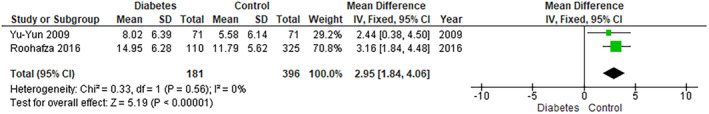
Dieting subscale EAT 26.

The pooled analysis showed a statistically significant association between the diabetes group and increased Dieting Subscale EAT 26 (MD = 2.95, 95% CI = 1.84–4.06, *p*‐value < 0.00001). We observed no significant heterogeneity among studies (*p* = 0.56, *I* ^2^ = 0%) (Figure 7).

#### 3.2.7 BITE Symptom Subscale

**FIGURE 8:**
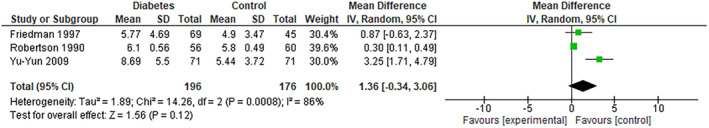
Bite symptom subscale.

The pooled analysis showed no statistically significant difference between the diabetes group and the control group (MD = 1.36, 95% CI = −0.34 to 3.06, *p*‐value = 0.12). We observed a significant heterogeneity among studies (*p* = 0.0008, *I* ^2^ = 86%) (Figure 8). So, we performed leave‐one‐out test by removing the study (Yu‐Yun 2009) and the heterogeneity was solved (*p* = 0.46, *I* ^2^ = 0%), and the pooled analysis showed a statistically significant association between the diabetes group and increased Bite Symptom Subscale (MD = 0.31, 95% CI = 0.12–0.50, *p*‐value = 0.001).

#### 3.2.8 BITE Severity Subscale

**FIGURE 9:**
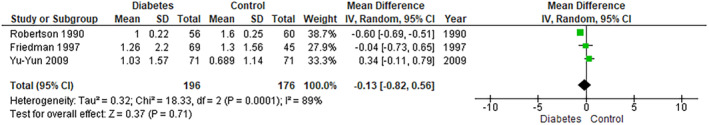
Bite severity subscale.

The pooled analysis showed no statistically significant difference between the diabetes group and the control group (MD = −0.13, 95% CI = −0.82 to 0.56, *p*‐value = 0.71). We observed a significant heterogeneity among studies (*p* = 0.0001, *I* ^2^ = 89%) (Figure 9). So, we performed leave‐one‐out test by removing the study (Robertson 1990) and the heterogeneity was solved (*p* = 0.37, *I* ^2^ = 0%), and also, the pooled analysis showed no statistically significant difference between the diabetes group and the control group (MD = 0.23, 95% CI = −0.15 to 0.60, *p*‐value = 0.24).

#### 3.2.9 Disordered Eating Behaviours

##### 3.2.9.1 Regular/Excessive Exercise

**FIGURE 10:**
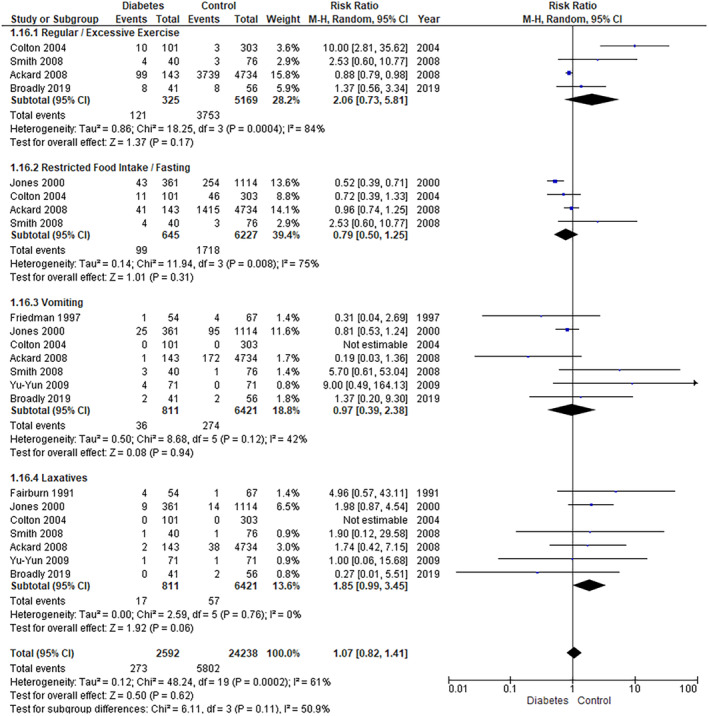
Behaviours 1.

The pooled analysis showed no statistically significant difference between the diabetes group and the control group (RR = 2.06, 95% CI = 0.73–5.81, *p*‐value = 0.17). We observed a significant heterogeneity among studies (*p* = 0.0004, *I* ^2^ = 84%) (Figure 10). So, we performed a leave‐one‐out test by removing the study (Colton 2004) and the heterogeneity was solved (*p* = 0.21, *I* ^2^ = 35%).

##### 3.2.9.2 Vomiting

The pooled analysis showed no statistically significant difference between the diabetes group and the control group (RR = 0.97, 95% CI = 0.39–2.38, *p*‐value = 0.94). We observed no significant heterogeneity among studies (*p* = 0.12, *I* ^2^ = 42%) (Figure 10).

##### 3.2.9.3 Laxatives

The pooled analysis showed no statistically significant difference between the diabetes group and the control group (RR = 1.85, 95% CI = 0.99–3.45, *p*‐value = 0.06). We observed no significant heterogeneity among studies (*p* = 0.76, *I* ^2^ = 0%) (Figure 10).

##### 3.2.9.4 Diuretic Misuse

**FIGURE 11:**
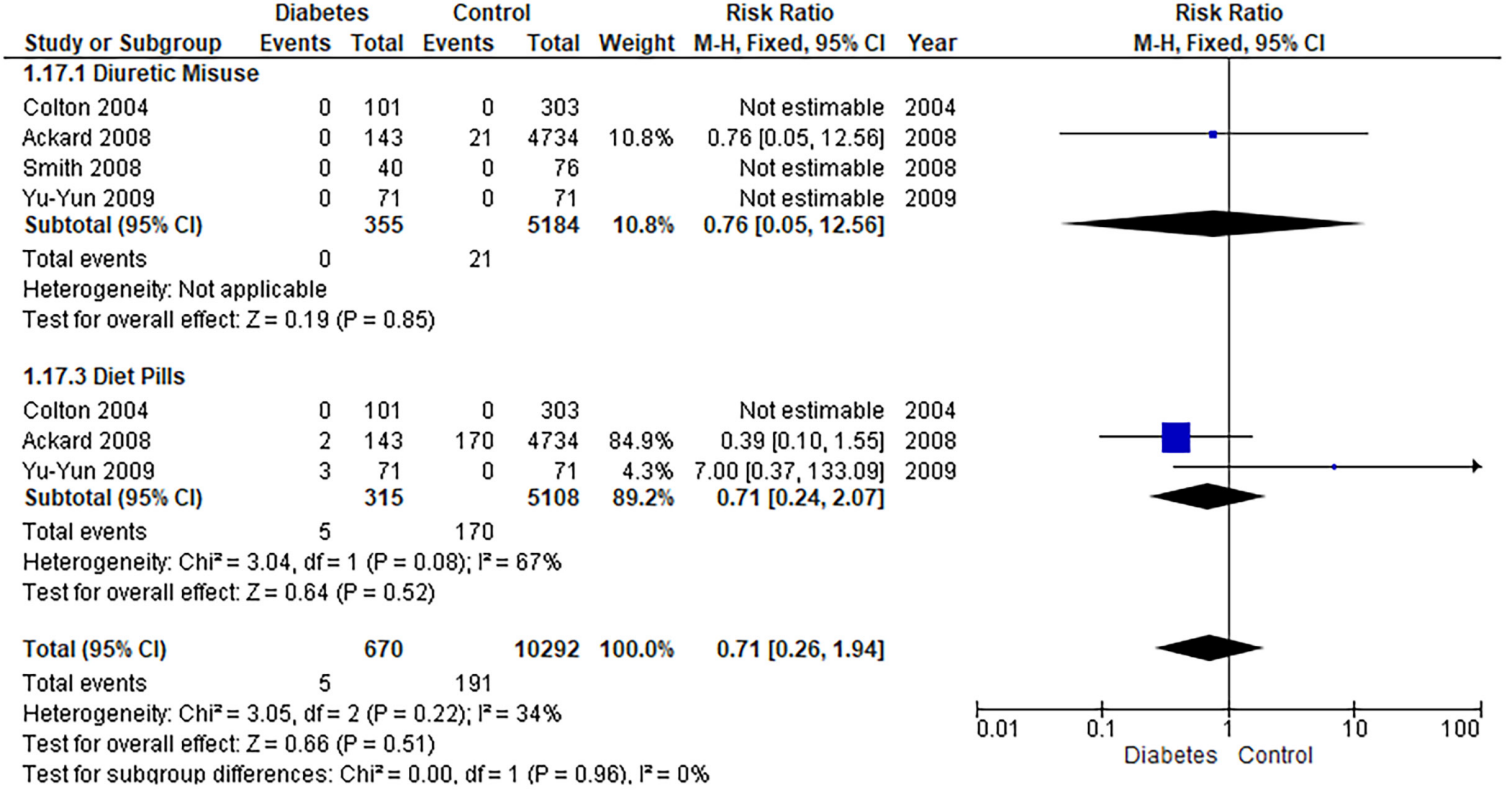
Behaviours 2.

The pooled analysis showed no statistically significant difference between the diabetes group and the control group (RR = 0.76, 95% CI = 0.05–12.56, *p*‐value = 0.85) (Figure 11).

##### 3.2.9.5 Diet Pills

The pooled analysis showed no statistically significant difference between the diabetes group and the control group (RR = 0.71, 95% CI = 0.24–2.07, *p*‐value = 0.52). We observed no significant heterogeneity among studies (*p* = 0.08, *I* ^2^ = 67%) (Figure 11).

##### 3.2.9.6 Insulin Omission and Misuse

**FIGURE 12:**
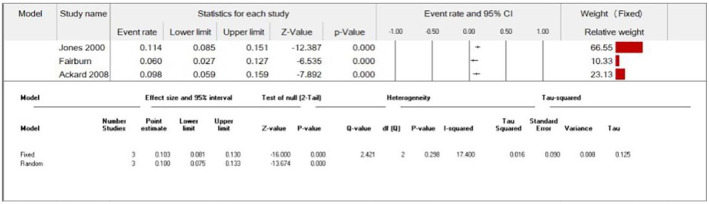
Insulin misuse/omission prevalence.

**FIGURE 13:**
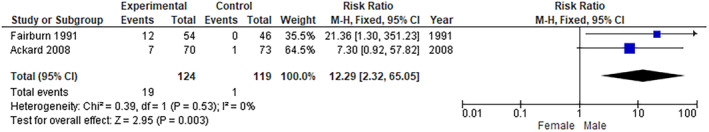
Insulin omission (females vs. males).

**FIGURE 14:**
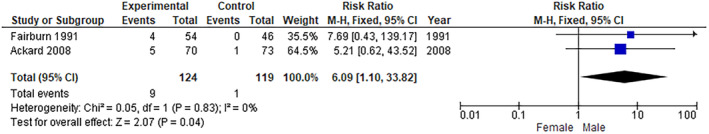
Insulin misuse (females vs. males).

The overall prevalence of insulin omission/misuse in our study sample was 10.3% (95% CI = 8.1–13) (Figure 12). Furthermore, the pooled analysis showed a statistically significant association between the female group and increased insulin omission compared with the male group (RR = 14.21, 95% CI = 2.66–76.04, *p*‐value = 0.002). We observed no significant heterogeneity among studies (*p* = 0.49, *I* ^2^ = 0%) (Figure 13). Additionally, the pooled analysis showed a statistically significant association between the female group and increased insulin misuse compared with the male group (RR = 6.51, 95% CI = 1.14–37.31, *p*‐value = 0.04). We observed no significant heterogeneity among studies (*p* = 0.83, *I* ^2^ = 0%) (Figure 14).

## 4 Discussion

Our analysis demonstrated that EDs were significantly prevalent among patients with T1DM compared with the nondiabetic individuals, specifically BN and binge eating, while no significant association was seen between T1DM and AN. The subgroup analysis, employing both DM‐Validated and Generic questionnaires for measuring EDs, revealed a statistically significant correlation between ED and T1DM. Additionally, the Eating Attitudes Test‐26 (EAT) showed a significant increase in the dieting and bulimia subscales among patients with T1DM. Furthermore, the Bulimic Investigatory Test, Edinburgh (BITE) showed a significant increase in the symptom subscale; however, no significant difference was detected between T1DM and controls in the severity subscale. Prevalence of insulin omission/misuse was 10.3%; diabetic females demonstrated a significantly higher risk of insulin omission and misuse than diabetic males. An analysis of other DEB showed insignificant associations between excessive exercise, dieting pills misuse, diuretics misuse and T1DM.

Commencing with an analysis of the subgroup data derived from DM‐Validated and Generic questionnaires assessing the prevalence of EDs, our research yielded intriguing results. Notably, we observed a significant association between the diagnosis of EDs, as indicated by both DM‐Validated and Generic questionnaires, and T1DM. These findings appear to diverge from the findings of Young et al., who posited that the prevalence of EDs is highly contingent upon the specific measurement tools employed. Young et al.'s research indicated that the effect size for EDs, as determined by DM‐validated questionnaires, exhibited statistical significance in relation to T1DM, whereas generic questionnaires did not. Their study suggested that the use of generic measures might lead to inflated prevalence estimates [3]. It is important to note that their study might have been constrained by an insufficient sample size as it was conducted in 2013 and did not account for the literature published thereafter, potentially affecting the statistical power of their analysis.

Based on our findings, we observed a significant correlation between EDs and T1DM. These results corroborate the conclusions drawn by Young et al., who arrived at a similar outcome. However, it is noteworthy that this association appears to be highly influenced by the method used to assess eating issues [3]. In contrast, Robertson and Rosenvinge and Troncone et al. did not discover a substantial link between EDs and T1DM. These studies had limitations, including a relatively small sample size. Moreover, Troncone et al. relied on parental evaluations, introducing potential bias [15, 18].

Our research demonstrates a significant connection between BN and T1DM, while AN does not exhibit the same association. These findings align with Mannucci et al.'s [2] previous meta‐analysis. In addition, García‐Reyna et al.'s [25] results found significant BN‐T1DM associations in men but not in women. Robertson and Rosenvinge [15] also found similar results concerning AN and T1DM. In contrast, Engström et al. [26] reported no significant BN‐T1DM association; however, their study had a higher number of BN cases in the T1DM group, with the main limitation being a female‐only study population. Colton et al.'s [27] research supports our findings regarding the significant link between binge eating and T1DM. Smith et al. [17] found that bulimia and binge EDs were more common in the diabetic group than in the control group.

Concerning the Dieting and Bulimia Subscales of the EAT 26 scoring and their relationship with T1DM, our results reveal a statistically significant connection. Similar results were reported by Roohafza et al. [23], while Pinar [29] found similar results, albeit only regarding the Dieting Subscale of the EAT 40 scoring, which is the precursor to the EAT 26 scoring [30]. Alice Hsu et al., however, reported a significant association between the Bulimia Subscale of the EAT 26 scoring and T1DM but found no association with the Dieting Subscale [24]. This discrepancy may be attributed to their relatively small sample size, which limited the ability to draw definitive conclusions, and the differences in educational levels between the control and T1DM groups, which may introduce the possibility of selection bias.

In terms of the BITE symptom and severity subscales, we did not find a significant association, but Alice Hsu et al. and Robertson and Rosenvinge reported mixed results. Alice Hsu et al. contradicted our findings regarding the BITE symptom subscale but aligned with us regarding the BITE severity subscale [24]. Robertson and Rosenvinge [15], on the contrary, reported no association between the BITE symptom subscale and T1DM, similar to our findings, but did find a significant connection with the BITE severity subscale. These mixed results may be attributed to both studies' limited statistical power due to small sample sizes. Additionally, Robertson and Rosenvinge [15] exclusively studied women, omitting male participants.

Additionally, on examining different DEBs, such as regular/excessive exercise, restricted food intake/fasting, vomiting, laxative use and insulin omission/misuse, we found no statistically significant associations except for insulin omission/misuse. Alice Hsu et al. also reported no significant association between vomiting, laxative use and T1DM [24]. Ackard et al. [20] yielded similar results across all eating behaviours analysed, highlighting a concerning percentage of T1DM participants who skipped or misused insulin doses. Furthermore, studies focusing on insulin misuse/omission corroborate our findings. Pinar [29] discovered a significant association between insulin misuse and T1DM, while Stancin et al. [31] reported that some female patients with diabetes intentionally omitted or underdosed insulin for weight reduction, even if they did not meet the criteria for an ED. Schober et al. [32] uncovered that nearly 30% of participants with T1DM intentionally manipulated insulin dosages and females being more at risk.

While a few studies contradict our eating behaviour analysis, such as Colton et al. [27], who found a significant association between excessive exercise and T1DM, and Smith et al. [17], who reported a higher prevalence of excessive exercise and self‐induced vomiting in the T1DM group, both studies had limitations, including low participation rates and potential recruitment bias from specialised clinics.

Given these findings, it is imperative to proactively screen and identify adolescents at risk of EDs. This can be achieved by employing evidence‐based tools such as the EAT 26 questionnaire analysed in the present study to accurately assess the presence of EDs/DEBs or the use of specific questionnaires for use in investigating ED in the diabetic population such as the Diagnostic Survey for Eating Disorders (DSED) and the Diabetes Eating Problems Survey (DEPS), which includes the assessment of Insulin manipulation, and should be performed by trained experienced healthcare provider interviewers. This proactive approach is especially vital during adolescence, as individuals in this age group may be inclined to hide their issues [33] or underreport behavioural problems related to their diabetes [34]. Moreover, we suggest the creation of comprehensive interventions tailored to this demographic, encompassing both diabetes and ED components that run concurrently.

It is noteworthy to highlight the results of Clery et al., who demonstrated that individuals with EDs associated with T1DM exhibit a less favourable response to conventional ED treatment and show limited improvement in their diabetes control. These findings suggest that individuals with T1DM‐related EDs may necessitate an alternative approach with a different level of intensity in their intervention [35].

### 4.1 Strengths and Limitations

This study has analysed the findings of 9079 individuals, which is more than triple the size of the meta‐analysis conducted by Mannucci et al. in 2005, with a sample size of 2592. Furthermore, their study only included females and their analysis of EDs was limited to AN and BN, whereas we analysed AN, BN, binge eating, Dieting Subscale EAT 26, Bulimia Subscale EAT 26, BITE symptom subscale, BITE severity subscale and different maladaptive behaviours practiced by patients with T1DM [2]. Another meta‐analysis conducted by Young et al. [3] didn't account for papers published after 2013.

It is important to note that ED entails avoidant/restrictive food intake disorder (ARFID) and binge eating disorder (BED); due to the lack of data, we could not run subgroup analyses on these EDs. Moreover, we could not analyse how the duration of diabetes, age of diabetics, race, socioeconomic status and BMI could potentially affect the association between T1DM and EDs. Further studies are warranted to explore the potential effects of these factors on the development of EDs among patients with T1DM.

## 5 Conclusion

Our study establishes a significant and clear connection between EDs and T1DM, particularly bulimia and binge eating, with T1DM. Moreover, female diabetics are at higher risk of insulin misuse/omission. Early proactive screening is essential, and tailored, comprehensive interventions combining diabetes and ED components are recommended for this population, with referral to a specialised psychiatrist.

## Author Contributions

**Yomna E. Dean:** Conceptualization (equal); data curation (equal); formal analysis (equal); methodology (equal); project administration (equal); supervision (equal); validation (equal); visualization (equal); writing – original draft (equal); writing – review and editing (equal). **Karam R. Motawea:** Formal analysis (equal); writing – original draft (equal). **Muaaz Aslam:** Data curation (equal); writing – original draft (equal). **Jose J. Loayza Pintado:** Data curation; writing. **Helen A. O. Popoola‐Samuel:** Data curation (equal); writing – original draft (equal). **Mohamed Salam:** Data curation (equal); writing – original draft (equal). **Prashant Obed Reddy Dundi:** Data curation (equal); writing – original draft (equal). **Webster Donaldy:** Data curation (equal); writing – original draft (equal). **Esraa M. Aledani:** Data curation (equal); writing – original draft (equal). **Zaineh Alqiqie:** Data curation (equal); writing – original draft (equal). **Nazia Sultana:** Data curation (equal); writing – original draft (equal). **Alaa Ramadan Hussein Mohamed:** Data curation (equal); writing – original draft (equal). **Amir Elalem:** Data curation (equal); writing – original draft (equal). **Sidra Tahreem Hashmi Syeda:** Data curation (equal); writing – original draft (equal). **Mai Saad Mohamed:** Formal analysis (equal). **Mazen W. Assal:** Data curation. **Nada M. Attia:** Data curation (equal); writing – original draft (equal). **Hanan Hagar:** Data curation (equal); writing – original draft (equal). **Heba Ahmed Abdelaziz:** Conceptualization (lead); writing – review and editing (equal). **Anuj Subedi:** Writing – review and editing (equal). **Areeg Elbahaie:** Data curation. Yusef Hazimeh: Supervision (equal); writing – review and editing (equal). **Hani Aiash:** Conceptualization (equal); investigation (equal); methodology (equal); project administration (equal); resources (equal); software (equal); supervision (lead); validation (equal); visualization (equal); writing – review and editing (equal).

## Ethics Statement

Not applicable.

## Conflicts of Interest

The authors declare no conflicts of interest.

## Supporting information


Appendix S1

## Data Availability

Data are available upon reasonable request to the corresponding author.
