# Supplementary material for: Association Between Type 1 Diabetes Mellitus and Eating Disorders: A Systematic Review and Meta‐Analysis
Source: Endocrinol Diabetes Metab. 2024 Apr 10;7(3):e473. doi: 10.1002/edm2.473 (PMC11005101; doi:10.1002/edm2.473)
Supplement: Supplementary file 1 — Appendix S1 [file EDM2-7-e473-s001.docx]

**PubMed (Jan 17^th^ 2023) (592)**

("Eating disorders" OR "disordered eating" OR "Disordered eating behaviors" OR "disordered eating attitudes" OR "feeding disorders" OR "appetite disorders" OR "Bulimia nervosa" OR "Bulimia" OR "binge eating" OR "Anorexia" OR "Anorexia nervosa" OR "irregular eating" OR "Irregular feeding" OR "body dysmorphic disorder" OR "body image disturbance" or dysmorphophobia OR dysmorphophobic OR BDD) AND ("Type 1 diabetes" OR "T1DM" OR "insulin dependent")

**Scopus (Jan 17^th^ 2023) (634)**

TITLE-ABS-KEY ( ( "Eating disorders" OR "disordered eating" OR "Disordered eating behaviors" OR "disordered eating attitudes" OR "feeding disorders" OR "appetite disorders" OR "Bulimia nervosa" OR "Bulimia" OR "binge eating" OR "Anorexia" OR "Anorexia nervosa" OR "irregular eating" OR "Irregular feeding" OR "body dysmorphic disorder" OR "body image disturbance" OR dysmorphophobia OR dysmorphophobic OR bdd ) AND ( "Type 1 diabetes" OR "T1DM" ) )

**Web of Science (17^th^ Jan 2023) (564)**

("Eating disorders" OR "disordered eating" OR "Disordered eating behaviors" OR "disordered eating attitudes" OR "feeding disorders" OR "appetite disorders" OR "Bulimia nervosa" OR "Bulimia" OR "binge eating" OR "Anorexia" OR "Anorexia nervosa" OR "irregular eating" OR "Irregular feeding" OR "body dysmorphic disorder" OR "body image disturbance" or dysmorphophobia OR dysmorphophobic OR BDD) AND ("Type 1 diabetes" OR "T1DM" OR "insulin dependent")
